# Evaluating Tumor Tissue Modified Viral (TTMV)-HPV DNA for the Early Detection of Anal Squamous Cell Carcinoma Recurrence

**DOI:** 10.3390/cancers17020174

**Published:** 2025-01-08

**Authors:** Rafi Kabarriti, Shane Lloyd, James Jabalee, Catherine Del Vecchio Fitz, Randa Tao, Tyler Slater, Corbin Jacobs, Sean Inocencio, Michael Rutenberg, Chance Matthiesen, Kasha Neff, Gene-Fu Liu, Tiffany M. Juarez, Stanley L. Liauw

**Affiliations:** 1 Montefiore Medical Center, Bronx, NY 10467, USA; rkabarri@montefiore.org; 2 Department of Radiation Oncology, University of Utah School of Medicine, Salt Lake City, UT 84113, USA; randa.tao@hci.utah.edu (R.T.); tyler.slater@hci.utah.edu (T.S.); 3 Naveris, Inc., Waltham, MA 02451, USA; jamie.jabalee@naveris.com (J.J.); catherine@naveris.com (C.D.V.F.); 4 Cancer Care Northwest, Spokane Valley, WA 99216, USA; corbin.jacobs@ccnw.net (C.J.); sean.inocencio@wsu.edu (S.I.); 5 Department of Radiation Oncology, Mayo Clinic, Jacksonville, FL 32224, USA; rutenberg.michael@mayo.edu; 6 Freeman Radiation Oncology, Joplin, MO 64804, USA; clmatthiesen@freemanhealth.com (C.M.); knneff@freemanhealth.com (K.N.); 7 Providence Mission Hospital, Mission Viejo, CA 92691, USA; gene-fu.liu@providence.org (G.-F.L.); tiffany.juarez@providence.org (T.M.J.); 8 Department of Radiation and Cellular Oncology, University of Chicago, Chicago, IL 60637, USA; sliauw@bsd.uchicago.edu

**Keywords:** anal cancer, TTMV-HPV DNA, cancer recurrence

## Abstract

Background: The incidence and mortality of anal squamous cell carcinoma (ASCC) are rising, with greater than 80% of cases linked to human papillomavirus (HPV), primarily HPV16. Post-treatment surveillance can be challenging due to the limitations of anoscopy, digital anal rectal exam (DARE), and imaging. Plasma tumor tissue modified viral (TTMV)-HPV DNA has shown strong sensitivity, specificity, and predictive value in detecting the recurrence of HPV-driven oropharyngeal cancer. Here, we investigate the ability of TTMV-HPV DNA for the early recurrence detection of ASCC. Methods: This retrospective clinical case series included 117 patients with HPV-driven ASCC across 7 U.S. centers, monitored with TTMV-HPV DNA during routine clinical care between March 2020 and June 2024. Physician-reported clinical data and biomarker testing data were combined to create a comprehensive, longitudinal dataset for evaluating test performance metrics. Results: Patients had a median age of 63 years and median post-diagnosis follow-up of 19 months. HPV status was primarily confirmed by TTMV-HPV DNA (52%) or p16 immunohistochemistry (39%). Of those tested for TTMV-HPV DNA pretreatment, 85% had a positive result. TTMV-HPV DNA clearance during or within three months post-treatment was associated with significantly better recurrence-free survival. The per-patient surveillance sensitivity, specificity, positive predictive value (PPV), and negative predictive value (NPV) were 82.8%, 98.4%, 96.0%, and 92.5%. Of 24 patients with a documented recurrence and a positive TTMV-HPV DNA test, the test was the first evidence of recurrence in 14 patients (58.3%), with a median lead time of 59 days (range: 10–536). TTMV-HPV DNA accurately resolved 94.3% of cases with indeterminate clinical findings. Conclusions: TTMV-HPV DNA testing provides a sensitive and specific approach for detecting patients with recurrent ASCC and resolving the status of patients with indeterminate clinical findings.

## Simple Summary

Anal squamous cell carcinoma (ASCC) is a rare cancer that is often driven by human papillomavirus and is increasing in incidence. Recurrence occurs in up to 30% of patients, making effective post-treatment surveillance both critical and challenging due to the limitations of clinical examination, anoscopy, and imaging. The release of fragmented tumor-associated viral DNA into the bloodstream provides a unique biomarker to aid in the diagnosis and surveillance of ASCC. This study evaluates circulating tumor tissue modified viral (TTMV)-HPV DNA in patients treated for HPV-driven ASCC. The findings demonstrate its utility in detecting patients with minimal residual, recurrent, and distant metastatic disease earlier than clinical and imaging approaches, providing insight into treatment response and resolving ambiguous clinical and imaging findings. By addressing gaps in traditional surveillance methods, TTMV-HPV DNA testing has the potential to improve outcomes for patients with ASCC.

## 1. Introduction

The incidence of human papillomavirus (HPV)-associated anal squamous cell carcinoma (ASCC) has risen significantly over recent decades. Particularly high rates of ASCC are seen among non-Hispanic White women aged 65 years or older in the United States, surpassing cervical cancer in this demographic [1]. Elevated rates of ASCC are also observed in populations with a higher prevalence of HIV, including men who have sex with men (MSM) [2]. Most ASCCs (>80%) are associated with HPV, particularly HPV16, a virus also implicated in most cases of cervical and oropharyngeal cancer [3,4]. As with other HPV-driven malignancies, ASCC presents unique challenges in its management and surveillance.

Current treatment varies significantly with the stage of the disease. While local resection may be considered for early-stage tumors, combined chemoradiation therapy (CRT) is the standard treatment for most patients, particularly in more advanced stages [4,5,6]. In cases of distant metastatic spread (Stage IV), treatment is primarily palliative, focusing on disease control and symptom relief through various modalities, including chemotherapy and radiation, sometimes supplemented with immunotherapy [4]. Despite effective initial responses to treatment, disease recurrence remains a significant risk, with up to 30% of patients experiencing locoregional failure or distant disease recurrence following CRT [6,7,8]. These observations underscore the need for accurate post-treatment surveillance to enable the early detection of recurrent patients, allowing for salvage treatment at the lowest tumor burden and minimizing disease- and treatment-related morbidity and mortality.

Current National Comprehensive Cancer Network (NCCN) guidelines for surveillance after primary treatment emphasize a multimodal approach that includes digital anal rectal examination (DARE), anoscopy, and radiographic imaging, particularly for high-risk patients [5]. Baseline treatment response is evaluated by DARE at 8–12 weeks post-treatment. For patients without any evidence of disease, returning to the clinic for DARE every 3–6 months for the first five years is recommended [5]. Anoscopy is recommended every 6–12 months to identify any local recurrence of suspicious lesions [5]. Patients with more advanced stages (Stage II–III) are at higher risk of recurrence, so annual radiographic imaging (such as CT, MRI, or PET scans) is advised for up to three years [5]. Despite these recommendations, patient access to high-resolution anoscopy and advanced imaging modalities can be highly limited by geography [9,10,11,12].

Circulating tumor DNA (ctDNA) has emerged as a pivotal biomarker in cancer management. Several previous studies have reported on the use of ctDNA in detecting ASCC [13,14,15,16,17,18,19,20]. Of particular importance, several studies have demonstrated the prognostic value of HPV ctDNA during ASCC surveillance. They found that residual ctDNA after the initial treatment of HPV-driven ASCC correlates with shorter disease-free and progression-free survival and reduced overall survival [13,14]. In the context of HPV-driven ASCC, differentiating HPV DNA from active viral infection and tumor-derived HPV DNA is critical [21]. The development of a specific and sensitive multianalyte droplet digital PCR (ddPCR) assay for detecting circulating cell-free tumor tissue modified viral (TTMV)-HPV DNA represents a significant advance in the detection of minimal residual disease (MRD). This novel biomarker, arising from the fragmentation of the integrated and/or episomal HPV DNA of malignant cells, has demonstrated strong analytical and clinical sensitivity and specificity in detecting HPV-driven cancers [22,23,24,25]. TTMV-HPV DNA could improve ASCC surveillance by enabling the non-invasive detection of MRD and recurrence and by offering a practical alternative for patients with limited access to specialized technology like anoscopy or advanced imaging.

The aims of this study were to assess the real-world performance of TTMV-HPV DNA in detecting recurrent HPV-driven ASCC under routine surveillance and to evaluate the relationship between detectable TTMV-HPV ctDNA after treatment by assessing recurrence-free survival. Previous studies in the context of HPV-driven oropharyngeal squamous cell carcinoma (OPSCC) have demonstrated equivalence or superior performance for TTMV-HPV DNA to detect MRD compared to standard-of-care surveillance methods [22,23]. We report both the per-test metrics, which evaluate the performance of individual assays, and per-patient metrics, which assess the cumulative impact across a patient’s surveillance period, providing complementary insights for clinical decision-making. TTMV-HPV DNA as a biomarker for ASCC MRD addresses the limitations of current surveillance methods by providing a more sensitive and specific clinical, procedure-independent, and broadly accessible method to detect recurrence. Current clinical methods often rely on symptomatic presentation, imaging, or the limited resources of high-resolution anoscopy, which can potentially delay the detection of recurrence.

## 2. Materials and Methods

### 2.1. Ethics Approval and Study Design

This retrospective clinical case series obtained Institutional Review Board (IRB) and data sharing approval at each of seven participating U.S. centers. Additionally, a waiver of written informed consent was granted by a central, independent IRB (WCG IRB; sponsor protocol number: NAV11042020), in alignment with recognized ethical guidelines. Participating patients had confirmed HPV-associated, pathology-proven ASCC and had a minimum of one TTMV-HPV DNA test result obtained between March 2020 and June 2024. Tests were collected during routine clinical practice and a pretreatment test was not required for study inclusion. Tumor HPV status was assessed at the discretion of the treating physician using p16 immunohistochemistry (IHC), a surrogate HPV marker, and/or directly using TTMV-HPV DNA testing (blood or tissue), HPV polymerase chain reaction (PCR), or HPV RNA in situ hybridization (ISH).

### 2.2. Data Collection and Annotation

Data were entered electronically by trained participants at each site into a central, de-identified database. Clinical data included demographics (sex as a biological variable, race and ethnicity, smoking status, HIV status, transplant status, date of initial diagnosis) and tumor information (location of the primary tumor, tumor staging [American Joint Committee on Cancer (AJCC), 8th edition [26]], HPV confirmation methodology and results, HPV strain [if known], and status of enrollment in a clinical trial), treatment history (start and end date, modality), clinical exam, imaging, and biopsy history (date, modality, and outcomes [coded as active disease, indeterminate/suspicious, or no evidence of disease], and anatomic site and HPV status of the biopsy, if known), and outcomes (alive or deceased, date of death or next follow-up).

### 2.3. TTMV-HPV DNA Testing

Peripheral blood was collected at the discretion of the treating physician during routine clinical care and tested for TTMV-HPV DNA using the NavDx^®^ assay (Naveris, Inc., Waltham, MA, USA). NavDx is a Clinical Laboratory Improvement Amendments (CLIA)-approved, analytically verified laboratory-developed test (LDT) that has been previously described [22,23,24,25]. Briefly, it is a multianalyte assay that utilizes ddPCR to analyze ctDNA fragmentation patterns. This assay specifically detects and quantifies circulating fragments of TTMV-HPV DNA from five high-risk HPV subtypes (16, 18, 31, 33, and 35) to identify patients with HPV-associated cancers. Results were reported qualitatively (positive, negative, or indeterminate) and quantitatively (as a TTMV-HPV DNA score). TTMV-HPV DNA test results were aligned with the patient’s treatment stage at the time of collection. TTMV-HPV DNA tests were considered true positives if they correlated with a positive exam, biopsy, or imaging study, and/or if the patient received salvage treatment. Tests collected before the start of initial curative-intent treatment were termed “pretreatment”, those performed any time between the start and end date of treatment were termed “during treatment”, and those carried out following the completion of treatment were termed “post-treatment”.

### 2.4. Statistical Analysis

Demographics, tumor characteristics, and TTMV-HPV DNA results were summarized descriptively. Test metrics were calculated for both the pretreatment (sensitivity) and surveillance settings (sensitivity, specificity, positive predictive value [PPV], and negative predictive value [NPV]). The clinical confirmation of recurrence was defined by an exam, imaging study, or biopsy showing active disease, or the initiation of salvage treatment. In the surveillance setting, a TTMV-HPV DNA test was considered true positive if the patient had a clinical confirmation of recurrence before, or any time after, a positive test; true negative if the patient did not have clinical confirmation of recurrence before or within three months of the testing date (negative tests without at least three months of clinical follow-up were censored); false positive if, at the time of death or the end of follow-up, there remained no clinical confirmation of recurrence and the patient had at least six months of clinical follow-up after a positive test; and false negative if the patient had clinical confirmation of recurrence before, or within three months of, the test. Indeterminate tests were treated as negative. Time-to-event analyses were conducted using the Kaplan–Meier method, with recurrence-free survival defined as the time from the end of initial treatment to the first recurrence. Patients who did not meet the endpoint were censored at the time of the last follow-up. Statistical tests were two-sided, and a *p*-value < 0.05 was considered significant.

## 3. Results

### 3.1. Study Population

Data were collected for 117 patients across seven U.S. institutions (median 13 patients/site, range: 4–40). Patients had a median age at diagnosis of 63 years (range: 36–91) and were predominantly female (85/117, 72.6%), White (85/117, 72.6%), and never-smokers (59/117, 50.4%). Of patients with known HIV and transplant status, most patients were HIV-negative (93/107, 86.9%) and had not received a transplant (114/116, 98.3%). For 106 patients, HPV status was confirmed by surrogate p16 IHC alone (84/117, 71.8%), p16 IHC in combination with HPV PCR or ISH (5/117, 4.3%), or by HPV PCR or ISH alone (4/117, 3.4%). For nine patients, a TTMV-HPV DNA analysis of tumor tissue was used to establish the HPV-positivity of the tumor and strain present. Fifteen patients (15/117, 12.8%) had documented HPV strains by unreported methodology. Of 81 patients for which HPV strain was known, most had HPV16 (74/81, 91.4%). Tumors were primarily located within the anal canal (113/117, 96.6%) and were primarily T2-T3 (76/117, 65.0%), N1 (73/117, 62.4%), and M0 (110/117, 94.0%). Most patients were initially treated with chemoradiotherapy (CRT) alone (101/117, 86.3%) or with adjuvant surgery (3/117, 2.6%), radiotherapy (3/117, 2.6%), chemotherapy (1/117, 0.85%), or immunotherapy (1/117, 0.85%). The remaining patients had radiotherapy alone (4/117, 3.4%), chemotherapy alone (2/117, 1.7%), or surgery alone (2/117, 1.7%). The median follow-up time, defined as the time from initial diagnosis to the last follow-up, across the entire cohort was 19 months (range: 0–83). For patients with one or more post-treatment tests, the median follow-up was 24 months (range: 6–83). Patient demographics are summarized in Table 1.

### 3.2. Patterns and Frequency of TTMV-HPV DNA Testing

The 117 patients underwent 368 total tests (median: 3, range: 1–11; Figure 1a). Testing was primarily post-treatment (267/368, 72.6%), followed by pretreatment (53/368, 14.4%), and during treatment (48/368, 13.0%). Pretreatment testing was carried out in 48 patients (41.0%), and during treatment testing was performed in 26 patients (22.2%). Nine patients received testing pretreatment, during treatment, and post-treatment.

**Figure 1:**
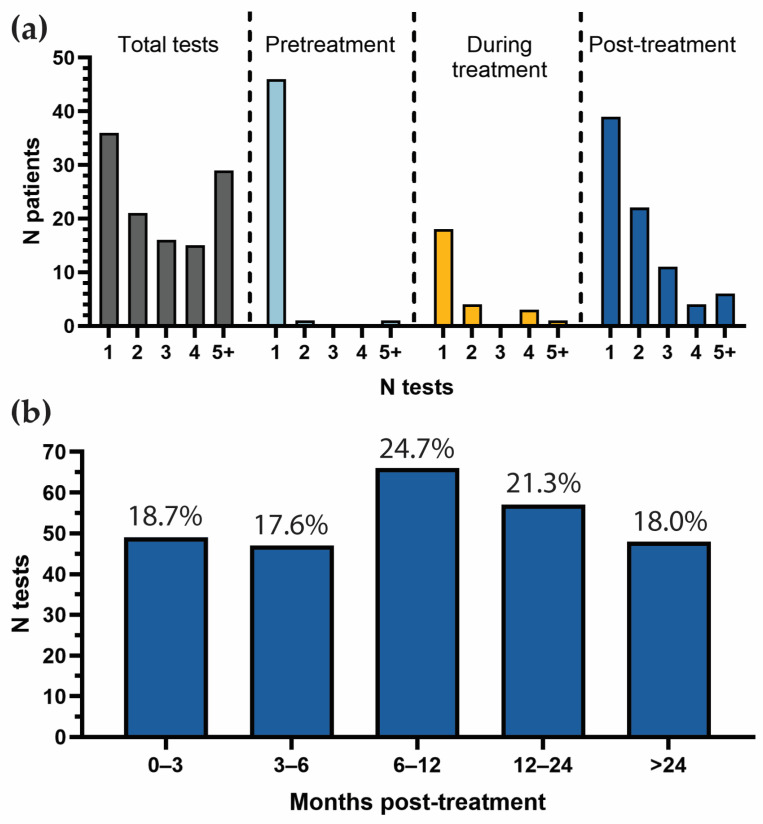
Distribution of TTMV-HPV DNA tests by treatment stage and timing. (**a**) Bar graph showing the distribution of patients according to the number of tests received. (**b**) Bar graph displaying the proportion of post-treatment tests by time interval, with intervals defined as left-inclusive.

For patients who received post-treatment surveillance testing (104/117, 88.9%), most received at least two tests (43/104, 41.3%) and a small cohort received five or more tests (6/104, 5.8%). The median number of post-treatment tests per patient was two (range: 1–9). Post-treatment tests were most commonly performed at 6–12 months post-treatment (66/267, 24.7%), followed by 12–24 months (57/267, 21.3%), 0–3 months (49/267, 18.0%), >24 months (48/267, 18.0%), and 3–6 months (47/267, 17.6%; Figure 1b). The median time between successive post-treatment tests was 3.7 months (range: 0.03–31.9), roughly in line with NCCN guidelines for post-treatment surveillance.

### 3.3. Baseline TTMV-HPV DNA Detectability

**Figure 2:**
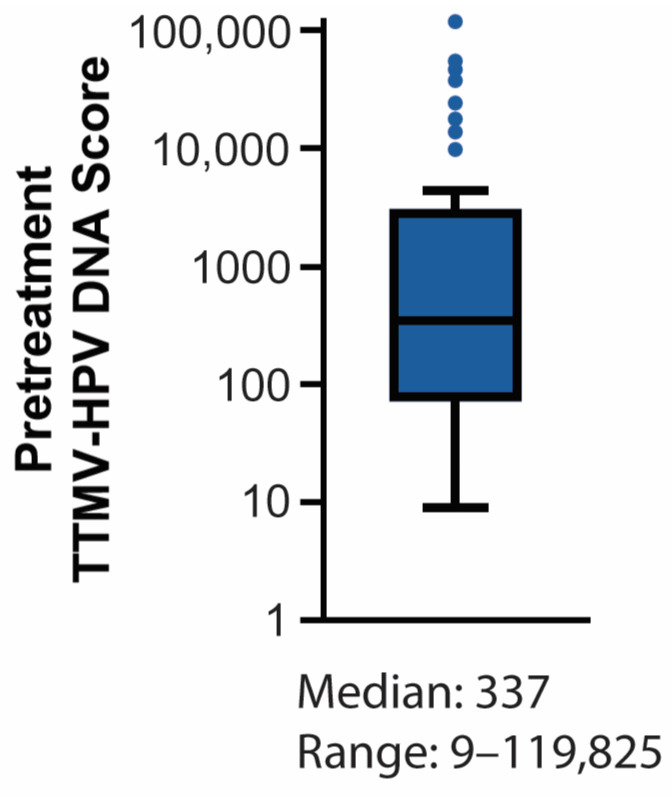
Pretreatment score distribution. Tukey-style boxplot illustrating the distribution of positive pretreatment test scores.

TTMV-HPV DNA was detectable at baseline in 85.4% of patients with a pretreatment test (41/48; 95% CI: 75.4–95.4; Table 2) with a median TTMV-HPV DNA score of 337 (range: 9–119,825; Figure 2). Pretreatment test sensitivity showed an increasing trend with nodal stage, though this was not statistically significant (Fisher’s exact test, *p* = 0.23). Pretreatment test sensitivity in patients without lymph node metastasis (stage N0) was 76.5% (13/17, 95% CI: 74.9–95.3), compared to 90.3% (28/31, 95% CI: 75.4–95.4) for patients with lymph node metastasis (stage N1; odds ratio 2.9).

### 3.4. Post-Treatment TTMV-HPV DNA Performance Metrics

Of 48 TTMV-HPV DNA tests conducted on 29 instances of post-treatment persistent or recurrent ASCC, 41 were positive and 7 were negative. The per-test sensitivity and PPV across all tests were 85.4% (41/48, 95% CI: 75.4–95.4) and 97.6% (41/42, 95% CI: 93.0–100.0), respectively (Table 3). Of the 29 recurrences, 24 were detected by TTMV-HPV DNA testing and 5 were not detected. The per-patient sensitivity and PPV were 82.8% (24/29, 95% CI: 69.0–96.5) and 96.0% (24/25, 95% CI: 88.3–100.0), respectively. All patients with relevant post-treatment testing were positive for p16 and/or HPV, and no patients had unconfirmed HPV status. Among five false negative patients, only one had a biopsy-confirmed recurrence, three went on to receive salvage therapy, and one received no further treatment. All patients with a false negative test had locoregional disease, except for one. This patient had M1 disease at baseline and had positive testing before their first and second salvage treatments (TTMV-HPV DNA score: 3615 and 5833, respectively, HPV16) before ultimately experiencing their third recurrence in the lung that was TTMV-HPV DNA negative. One additional false negative test was censored, as the patient had a biopsy carried out 41 days prior to testing, which may have removed the lesion and altered the level of TTMV-HPV DNA. Further censoring of the unconfirmed recurrences (those without either a biopsy or salvage therapy) yielded a per-test and per-patient sensitivity of 91.1% (41/45, 95% CI: 82.8–99.4) and 85.7% (24/28, 95% CI: 72.8–98.7), respectively.

A sub-analysis of only those patients with baseline positive TTMV-HPV DNA testing yields a total of 18 positive post-treatment tests across 9 patients. Here, the per-test sensitivity and PPV were 90.0% (18/20, 95% CI: 76.9–100.0) and 100% (18/18) and the per-patient sensitivity and PPV were 90% (9/10, 95% CI: 71.4–100.0) and 100% (9/9), respectively. The censoring of false negative tests lacking biopsy confirmation results in a per-test and per-patient sensitivity of 94.7% (18/19, 95% CI: 84.7–100.0) and 90.0% (9/10, 95% CI: 71.4–100.0), respectively.

Among 67 patients with no recurrent ASCC, there were 141 negative post-treatment tests. This yielded a per-test specificity and NPV of 99.3% (134/135, 95% CI: 97.9–100.0) and 95.0% (134/141, 95% CI: 91.6–98.7) and per-patient specificity and NPV of 98.4% (62/63, 95% CI: 95.3–100.0) and 92.5% (62/67, 95% CI: 86.2–98.8), respectively (Table 3). The censoring of the recurrences that were not confirmed (either by biopsy or by subsequent salvage treatment) yields a per-test and per-patient NPV of 97.1% (134/138, 95% CI: 94.3–99.9) and 93.9% (62/66, 95% CI: 88.2–99.7).

A sub-analysis of the 43 post-treatment negative tests among the 19 patients with a pre-treatment positive TTMV-HPV DNA test revealed a per-test specificity and NPV of 100% (39/39) and 95.3% (41/43, 95% CI: 89.1–100.0), respectively. Among these 19 patients, the per-patient specificity and NPV were 100% (17/17) and 94.7% (18/19, 95% CI: 84.7–100.0), respectively. The censoring of false negative tests lacking biopsy confirmation results in a per-test and per-patient NPV of 97.6% (41/42, 95% CI: 92.7–100.0) and 94.7% (18/19, 95% CI: 84.7–100.0), respectively.

### 3.5. Correlation with Clinical Recurrence

Of the 117 total patients, 104 patients (88.9%) had TTMV-HPV DNA testing post-treatment. Of these, 27 patients (26.0%) recurred with a median disease-free interval of six months, including local failure in 14 patients (14/27, 51.9%) and distant metastasis in 13 patients (13/27, 48.1%). There were a total of 268 post-treatment TTMV-HPV DNA tests across these 104 patients. Patients had a median of two post-treatment tests (range: 1–9). Most patients with post-treatment testing had at least one negative post-treatment test (90/104, 86.5%), and 56/104 (53.8%) patients had multiple negative tests (range: 1–9). The median time between consecutive negative post-treatment tests was 4.1 months (range: 0.03–31.9). The median time from a patient’s last negative post-treatment test and subsequent clinical recurrence detection was three months (range: 1.3–14.3). A patient’s first positive post-treatment test was sometimes preceded by one or more negative post-treatment tests by a median lead time of 4.9 months (range: 1.8–13.8).

**Figure 3:**
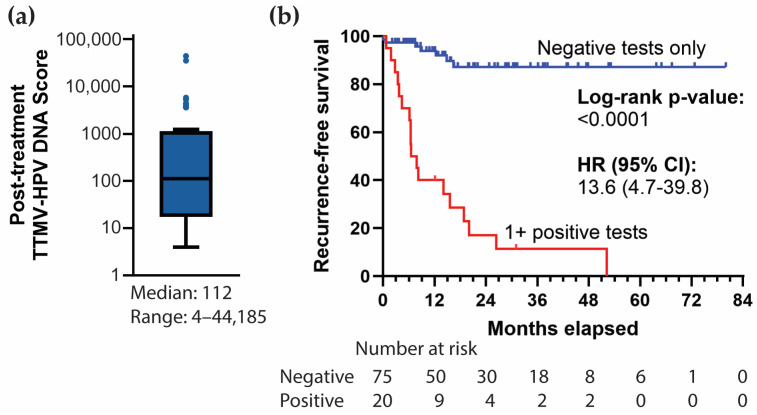
Post-treatment TTMV-HPV DNA score distribution and effect of positive testing on recurrence-free survival. (**a**) Tukey-style boxplot illustrating the distribution of positive post-treatment TTMV-HPV DNA scores. (**b**) Survival curve in which patients with post-treatment positive testing are separated based on whether they have positive testing during post-treatment. Kaplan–Meier method with two-sided log-rank test. CI: Confidence interval. HR: Hazard ratio.

**Figure 4:**
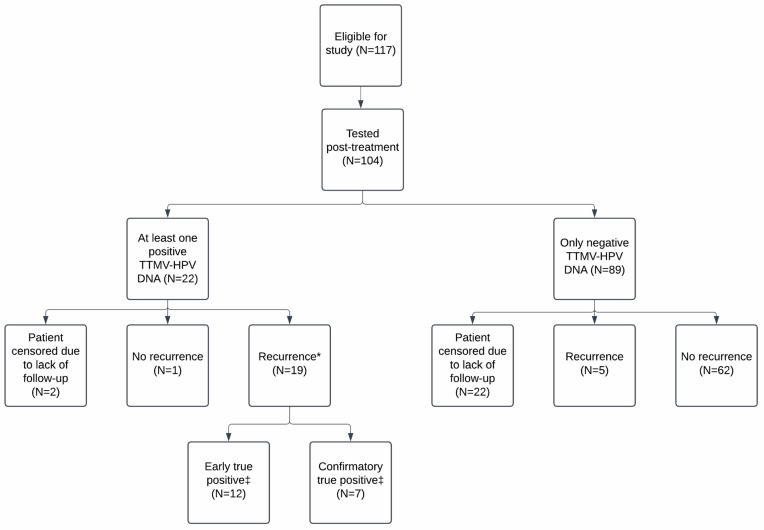
CONSORT diagram of patient inclusion, testing, and recurrence. * There were a total of 24 recurrences across 19 patients with positive TTMV-HPV DNA testing. ‡ Across 24 total recurrences, 14 were associated with early true positive testing and 10 with confirmatory true positive testing.

**Figure 5:**
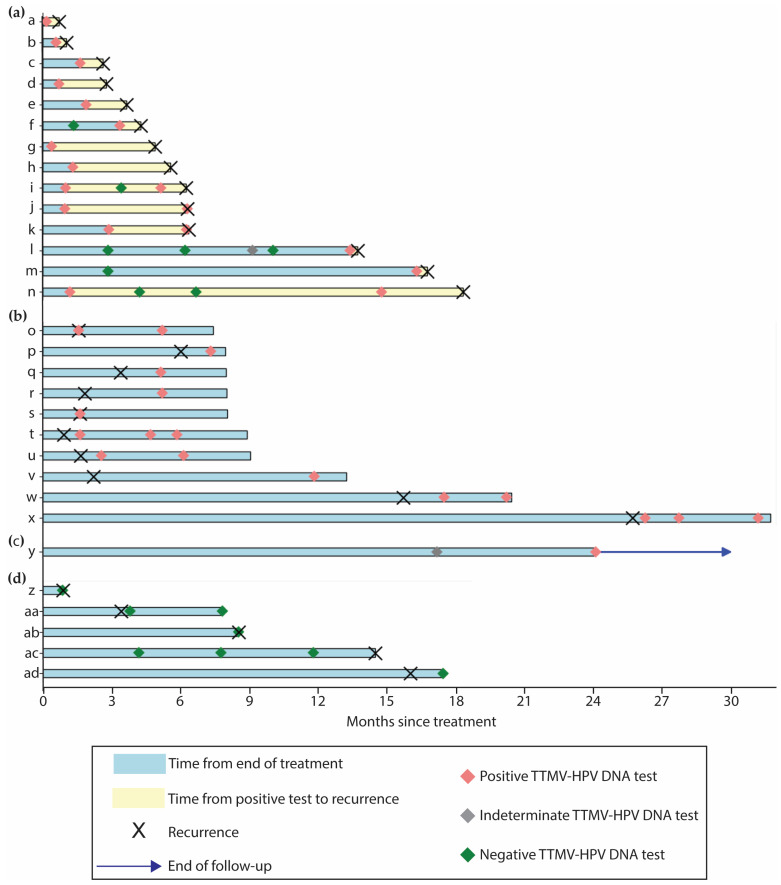
Swimmer plots for patients with clinically confirmed recurrence or positive post-treatment TTMV-HPV DNA testing. (**a**) Early true positives (*n* = 14). TTMV-HPV DNA was the first sign of recurrence. (**b**) Confirmatory true positives (*n* = 10). Positive TTMV-HPV DNA was coincident with or following clinical recurrence detection. (**c**) False positives (*n* = 1). Positive TTMV-HPV DNA in the absence of clinical recurrence detection. (**d**) False negatives (*n* = 5). TTMV-HPV DNA was negative and occurred either after, or within three months before, clinical recurrence detection. Only patient “ac” had a biopsy-confirmed HPV-positive recurrence.

A positive post-treatment TTMV-HPV DNA score was reported for 22 patients (22/104, 21.2%) across 44 tests with a median TTMV-HPV DNA score of 112 (range: 4–44,185; Figure 3a). Two positive tests from two patients were censored due to a lack of clinical follow-up (Figure 4). A total of 24 confirmed recurrences across 19 patients were associated with a positive TTMV-HPV DNA test. Notably, positive testing occurred before clinical recurrence detection in 14 instances (14/24, 58.3%), with a median lead-time of 59 days (range: 10–536; Figure 5a). In two instances (2/24, 8.3%), positive testing coincided with clinical recurrence detection; in eight instances (8/24, 33.3%), positive testing occurred after clinical recurrence detection (Figure 5b). For these 10 cases, no lead time was assessed as there was no testing prior to clinical recurrence detection. One patient with a positive test had no clinical evidence of recurrence (Figure 5c). The patient had an indeterminate post-treatment test (TTMV-HPV DNA score: 2, HPV16) followed by a positive test (TTMV-HPV DNA score: 13, HPV16) and has since had 189 days of follow-up without recurrence, which included a clinical exam and an MRI but no further TTMV-HPV DNA testing. Patients with one or more positive post-treatment tests had significantly worse recurrence-free survival than patients with negative testing throughout post-treatment (log-rank test, *p* < 0.0001; Figure 3b).

### 3.6. Resolution of Positive Baseline TTMV-HPV DNA

**Figure 6:**
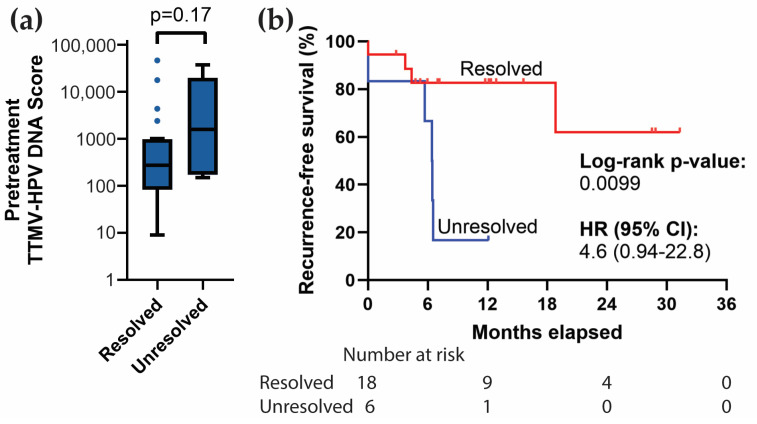
Baseline TTMV-HPV DNA resolution and recurrence-free survival. (**a**) Tukey-style boxplot comparing pretreatment TTMV-HPV DNA scores for patients whose test resolves vs. those whose tests remain unresolved by three months post-treatment. (**b**) Survival curve in which patients with pretreatment positive testing are separated based on whether their test score resolves during or within three months after initial treatment. Kaplan–Meier method with two-sided log-rank test. CI: Confidence interval. HR: Hazard ratio. ns: not significant.

Of the 41 patients with positive pretreatment TTMV-HPV DNA scores, 25 (25/41, 61.0%) were tested again during and/or within three months after initial definitive treatment. Among these, 19/25 (76.0%) showed a resolution of their detectable TTMV-HPV DNA scores. Eighteen patients received CRT and one patient received chemotherapy alone. Pretreatment TTMV-HPV DNA scores did not differ significantly between patients whose test became negative versus those who continued to have a positive test (Mann–Whitney U-test, U = 41.0, *p* = 0.17; Figure 6a). Patients whose tests became negative had significantly better recurrence-free survival than patients whose tests remained positive (log-rank test, *p* = 0.0099; Figure 6b).

### 3.7. Resolution of Clinically Indeterminate Findings (CIFs)

Clinically indeterminate findings (CIFs) occur when clinical exams and/or imaging studies yield equivocal results. Across 52 patients, we identified 143 post-treatment CIFs. Of these, 57 (57/143, 39.9%) were associated with a TTMV-HPV DNA test performed within three months and in patients who were followed clinically for at least three additional months.

To ensure that each TTMV-HPV DNA test was evaluated only once, CIF events temporally matching the same TTMV-HPV DNA test were grouped together, resulting in 35 instances of CIF(s) with a matched TTMV-HPV DNA test for analysis. Among these, the TTMV-HPV DNA test correctly predicted recurrence status in 33 cases (33/35, 94.3%). Five tests correctly predicted recurrence, twenty-eight correctly ruled out recurrence, and two incorrectly ruled out recurrence.

In contrast to the frequency of post-treatment CIFs (*n* = 141), indeterminate TTMV-HPV DNA tests were rare. Only five were reported, representing 1.9% of post-treatment tests (5/267).

## 4. Discussion

This study highlights the potential of TTMV-HPV DNA as a useful and significant biomarker for detecting ASCC recurrence, offering a surveillance tool with a per-patient sensitivity of 82.8% and a specificity of 98.4%. These metrics are comparable to its application in oropharyngeal squamous cell carcinoma (OPSCC), where it has facilitated the detection of MRD, recurrent, and distant metastatic disease [22,23]. The per-patient positive predictive value (PPV) of 96.0% and negative predictive value (NPV) of 92.5% highlight the clinical relevance of TTMV-HPV DNA testing. These metrics suggest that most patients with negative results can confidently be considered recurrence-free, potentially minimizing unnecessary interventions. Our findings, based on a median follow-up of two years within a multi-institutional cohort, demonstrate the potential of TTMV-HPV DNA to detect recurrences during critical periods when they are most likely to occur.

Notably, recurrent disease was confirmed in 24 instances across 19 patients who had a positive TTMV-HPV DNA test. In 58.3% of these cases, the positive test was the first evidence of recurrence, providing a median lead time of 59 days (range: 10–536). In the other cases, no TTMV-HPV DNA testing was conducted before clinical recurrence detection. This suggests that TTMV-HPV DNA may provide an early warning of recurrence, potentially preceding clinical symptoms or imaging detection.

During post-treatment surveillance, 21.2% of patients had at least one positive TTMV-HPV DNA test, with a PPV of 96.0%. This PPV is within the expected range for recurrence detection, corroborating previous research on the effectiveness of TTMV-HPV DNA in detecting occult disease recurrence in HPV-driven oropharyngeal carcinomas and extending its potential utility to anal carcinoma [22,23]. The sensitivity and PPV underscore the need for careful patient counseling and the development of clinical action thresholds that consider test positivity alongside clinical context and patient history.

The test’s NPV and specificity are particularly beneficial in post-treatment settings, where negative test results can strongly indicate the absence of disease. This feature could enable more personalized follow-up schedules, potentially extending the intervals between tests for patients with consecutive negative results, thus reducing both psychological and financial burdens.

The integration of TTMV-HPV DNA testing into clinical pathways shows promise. Additional research is required to assess its utility relative to standard-of-care approaches. For example, its application immediately post-treatment or during routine follow-up could be optimized according to test performance in various clinical scenarios. However, the detection of false negatives—where patients later exhibited recurrence verified to be HPV-positive by biopsy—underscores the need to continue refining the test’s sensitivity and our understanding of the dynamics of TTMV-HPV DNA.

Equivocal clinical examinations and diagnostic tests can place a significant psychological toll on patients, leading to increased anxiety [27,28,29]. In this study, TTMV-HPV DNA testing demonstrated 94.3% accuracy in resolving clinically indeterminate findings (CIFs), providing a more definitive diagnostic tool that may reduce the uncertainty associated with traditional imaging techniques and facilitate timely therapeutic intervention. These findings align with previous research on TTMV-HPV DNA’s effectiveness in resolving CIFs in oropharyngeal cancer [16].

This study has strengths and limitations that should be considered when interpreting the findings. Anal cancer is a relatively rare disease, with an incidence of approximately 10,000 cases per year in the USA [6,22]. While our cohort size may appear small, it represents approximately 1% of the annual incidence. The patients were unselected and represented a broad population treated at seven geographically dispersed sites and tested with NavDx, enhancing the generalizability of findings despite the retrospective design.

Limitations of the study include the lack of standardized timing or frequency of testing across patients, introducing variability in assessing the interval between positive test results and the subsequent detection of recurrence by clinical examination or imaging. Additionally, some positive tests coincided with or were performed shortly after clinical recurrence detection. While this provided data for evaluating clinical validity metrics such as sensitivity, specificity, NPV, and PPV, it limited the ability to assess an earlier detection interval. Further, HPV identification using the indirect method of p16 immunohistochemistry may lead to a misclassification of patients compared to the definitive HPV association using PCR or ISH. This has been reported, with agreement rates ranging from 79% to 95% [30,31,32]. A patient who is HPV-negative on PCR or ISH but demonstrates positive p16 staining could be incorrectly classified as a “false negative” at baseline or recurrence if only p16 results are considered, potentially affecting the accuracy of the TTMV-HPV DNA test interpretations.

Furthermore, although positive test results are expected to prompt clinicians to quickly confirm recurrences using clinical examination, imaging, or biopsy—based on the patterns observed in the test’s widespread use for recurrent HPV-driven oropharyngeal cancer—this behavior could not be directly documented in a retrospective study. Future studies may help refine the earlier detection interval. However, experience with OPSCC suggests that clinicians, aware of the test’s high PPV, are often reluctant to delay confirming recurrent disease. This makes such studies difficult to propose and challenging from an ethics and informed consent perspective.

Despite these limitations, the purpose of the test is to address gaps in current surveillance methods, such as DARE and imaging, which our study found to frequently yield indeterminate findings that require additional workup. A plasma test evaluating TTMV-HPV DNA offers universal accessibility, potentially improving compliance with ASCC surveillance guidelines by eliminating the need for patients to travel to specialized institutions or access limited specialty resources such as high-resolution anoscopy or specialized imaging. However, the impact of the test on patient compliance with surveillance was not evaluated in this study. Additional retrospective and prospective studies are in development or are underway.

## 5. Conclusions

In conclusion, integrating TTMV-HPV DNA testing into routine post-treatment surveillance holds potential to improve the management of HPV-driven ASCC. As a blood test, it is readily accessible, promoting health equity by reducing barriers to adherence with guideline-specified surveillance intervals. This approach may refine current protocols and enhance patient outcomes by facilitating earlier detection and treatment of recurrences.

## Figures and Tables

**Table 1:** Patient characteristics.

Characteristic	*n* = 117 (%)
Median age, years (range)	63 (36–91)
Biological sex	
Female	85 (72.6)
Male	32 (27.4)
Race and ethnicity	
White	85 (72.6)
Black or African American	12 (10.3)
Other	15 (12.8)
Preferred not to answer	5 (4.3)
Smoking status	
Never	59 (50.4)
Former (≥10 pack years)	32 (27.4)
Current	25 (21.4)
HIV status	
Negative	93 (79.5)
Positive	14 (12.0)
Unknown	10 (8.5)
Transplant status	
Not a transplant patient	114 (97.4)
Transplant patient	2 (1.7)
Unknown	1 (0.9)
Site of primary disease	
Anal canal, no pelvic involvement	74 (63.2)
Anal canal with pelvic involvement ^A^	39 (33.3)
Anal/perianal skin	3 (2.6)
Rectum	1 (0.9)
HPV reference testing method ^B^	
p16 (by IHC) only	84 (71.8)
p16 IHC and HPV PCR or ISH	5 (4.3)
HPV PCR or ISH only	4 (3.4)
TTMV-HPV DNA (FFPE tissue)	9 (7.7)
Unreported methodology	15 (12.8)
HPV subtype ^C^	
16	74 (92.5)
16/18	1 (0.9)
18	3 (2.6)
33	2 (1.7)
Initial primary tumor staging ^D^	
T0	1 (0.85)
T1	19 (16.2)
T2	44 (37.6)
T3	32 (27.4)
T4	21 (17.9)
Nodal status at baseline ^D^	
N0	44 (37.6)
N1	73 (62.4)
Distant metastasis at baseline ^D^	
M0	110 (94.0)
M1	7 (6.0)
Cancer stage	
I	12 (10.3)
II	30 (25.6)
III	68 (58.1)
IV	7 (6.0)
Initial treatment modality	
CRT	106 (90.6)
Radiation	4 (3.4)
Surgery, CRT	3 (2.6)
Chemotherapy	2 (1.7)
Surgery	2 (1.7)

^A^ Anal cancer with pelvic involvement refers to tumors that have extended beyond the anal canal and infiltrated neighboring pelvic structures, such as the rectum, colon, vagina, or pelvic sidewall. ^B^ No patient was p16 negative and confirmatory HPV ISH or PCR positive. ^C^ HPV subtype was only available for those patients with a positive TTMV-HPV DNA test (*n* = 80). ^D^ According to the American Joint Committee on Cancer, 8th Edition [26]. CRT, chemoradiation therapy.

**Table 2:** Pretreatment sensitivity of patients with confirmed HPV-driven disease.

Cohort	True Positives	False Negatives	Sensitivity	95% CI
Per-patient	41	7	85.4	75.4–95.4

**Table 3:** Post-treatment metrics of TTMV-HPV DNA.

	**Per-Test Accuracy Measures**		
	Disease “+”	Disease “−”	Predictive value ^A^
Test positive	41	1	PPV = 97.6% (93.0–100)
Test negative	7	134	NPV = 95.0% (91.5–98.6)
Sensitivity and specificity	Sens. = 85.4% (75.4–95.4)	Spec. = 99.3% (97.8–100)	
	**Per-Patient Accuracy Measures**		
Test positive	24	1	PPV = 96.0% (88.3–100)
Test negative	5 ^A,B^	62	NPV = 92.5% (86.2–98.8)
Sensitivity and specificity	Sens. = 82.8% (69.0–96.5)	Spec. = 98.4% (95.3–100)	

^A^ Only one of the five patients with a false negative test had pretreatment or baseline testing results. Therefore, baseline detectability of TTMV-HPV DNA was not confirmed among four of the five patients. ^B^ One patient was censored, as they had a biopsy preceding testing that may have altered TTMV-HPV DNA detectability. The 95% confidence intervals (95% CI) are shown in parentheses for respective values. Sens., sensitivity; Spec., specificity; PPV, positive predictive value; NPV, negative predictive value.

## Data Availability

The data supporting this study’s findings are available from the corresponding author upon reasonable request and subject to institutional review and data-sharing agreements.
